# Survival Trends for Uterine Sarcomas from a Tertiary Center: The Oxford Experience

**DOI:** 10.3390/diseases12090200

**Published:** 2024-09-02

**Authors:** Aakriti Aggarwal, Federico Ferrari, Andreas Zouridis, Sean Kehoe, Sarah Pratap, Elisa Gozzini, Hooman Soleymani Majd

**Affiliations:** 1 Department of Obstetrics and Gynecology, John Radcliffe Hospital, Oxford University Hospitals, NHS Foundation Trust, Oxford OX3 9DU, UK; aakritib21@gmail.com; 2 Department of Clinical and Experimental Sciences, Obstetrics and Gynecology, University of Brescia, 25100 Brescia, Italy; 3 Department of Gynaecological Oncology, Churchill Hospital, Oxford University Hospitals, NHS Foundation Trust, Oxford OX3 7LE, UKsean.kehoe@ouh.nhs.uk (S.K.); 4 Department of Clinical Oncology, Churchill Hospital, Oxford University Hospitals, NHS Foundation Trust, Oxford OX3 7LE, UK; sarah.pratap@ouh.nhs.uk; 5 Nuffield Department of Women’s Reproductive Health, Medical Sciences Division, Oxford University, Oxford OX3 9DU, UK

**Keywords:** uterine sarcoma, uterine leiomyosarcoma, endometrial stromal sarcoma, adenosarcoma, sarcoma, survival

## Abstract

**Simple Summary:**

We analyzed the clinicopathological characteristics, management, and survival trends in women diagnosed with uterine sarcoma at our tertiary institute over ten years. We studied the recurrence trends and their management, with a further focus on patterns of the follow-up. This study generates meaningful real-world data to help offer more insight into these rare gynecological cancers.

**Abstract:**

Uterine sarcomas are rare and aggressive gynecological malignancies. We evaluated the epidemiology, treatment outcomes and survival rates in uterine sarcoma patients managed in our institute. The medical records of women with histology proven uterine sarcoma, including leiomyosarcoma (LMS), adenosarcoma (ADS), and endometrial stromal sarcoma (ESS), treated at our institution from February 2010 to February 2022, were analyzed. In total, 41 patients were identified. In detail, LMS, ADS, and high-grade and low-grade ESS were identified, respectively, in 60.9%, 19.5%, 12.1%, and 7.3% of the cases. The majority of women affected by LMS (72%) underwent primary surgery and 40% of them also received adjuvant chemotherapy. A surgical approach was the preferred mode of treatment in 83% of the recurrences. The median OS (overall survival), DFS (disease free survival), and PFS (progression free survival) for the LMS group were 25, 44.5, and 8 months, respectively. The 5-year survival rates for LMS, ADS, and ESS groups were 30.5%, 100% and 37.5%, respectively. The 5-year survival for LMS was found to be significantly worse than for other histology types (*p* = 0.016). Our study provides valuable data for the evaluation of treatment strategies and survival trends among these rare cancers. The management and follow-up planning of each subtype requires a thorough patient-focused multidisciplinary discussion.

## 1. Introduction

Uterine sarcomas are a heterogenous group of rare malignant tumors, constituting 1–3% of all gynecological cancers and 3–7% of all malignant uterine tumors [1,2,3,4]. They are histopathologically different and highly invasive tumors that originate from the endometrial connective tissue or myometrium [5]. The commonest histological subtypes include uterine leiomyosarcoma (LMS), adenosarcoma (ADS), endometrial stromal sarcoma (ESS), and undifferentiated uterine sarcoma (UUS). The 2014 World Health Organization (WHO) classification divides uterine sarcoma into endometrial stromal sarcoma (ESS) classified as low-grade (LG-) and high-grade (HG-) ESS [6]. The latest 2020 WHO classification maintained this classification and further expanded on the classification characteristics, including the morphologic and immunophenotypic profiles associated with *YWHAE*::*NUTM2A/B* fusions, *BCOR* internal tandem duplications, and *ZC3H7B*::*BCOR* fusions in these tumors [7,8]. Many of these tumors have poor outcomes because of their inherently aggressive tumor biology, late-stage presentation, and high risk of recurrence [9].

Pelvic pain, irregular uterine bleeding, and the bulking effect from uterine masses are the symptoms of uterine sarcomas. Typically, the diagnosis is made after surgery for benign leiomyoma or is signaled by the advanced spread of the disease. ADS has a lower risk of spread and later recurrence compared to other uterine sarcomas [10]. Imaging can help to establish the suspicion of uterine sarcoma preoperatively, identifying solid masses, with irregular echogenicity with occasionally irregular cystic areas, and very rarely, fan-shaped shadowing, which is usually highly vascularized [11,12]. Surgical intervention is regarded as the cornerstone of treatment, with other modalities—such as chemotherapy—demonstrating limited results [13,14,15]. There is a paucity of clear guidance regarding the follow-up regimens, and recurrences are usually managed surgically as much as possible [16].

Over the past few decades, chemotherapy has replaced radiation therapy as the adjuvant treatment, although survival has still been unchanged. The relationships between prognosis and patient age, clinical stage, tumor size, and vascular invasion have not been consistently highlighted in the literature [17].

This study aims to examine the clinicopathological characteristics and survival trends in women diagnosed and managed with uterine sarcomas. We also studied the follow-up regimens used to monitor these patients after the primary intervention and evaluated the management of the recurrences.

## 2. Materials and Methods

### 2.1. Setting and Considerations

This was a retrospective study of patients diagnosed with and managed for uterine sarcoma at our institute. The inclusion criterion was a histopathological diagnosis of uterine sarcoma. We included all patients with LMS, ADS, and ESS treated across the Thames Valley Cancer Alliance Network between February 2010 and February 2022. It should be noted that uterine carcinosarcoma (also known as mixed Mullerian tumor) was previously considered a subtype of uterine sarcoma but has been reclassified as an epithelial uterine tumor and therefore was excluded from our study. We did not come across any case of undifferentiated uterine sarcoma during the study period. Oxford University Hospitals National Health Service (NHS) Trust acted as the tertiary center for all these cases. All data were extracted retrospectively from electronic records of women in the context of service evaluation for the management of patients with uterine sarcoma. The service evaluation protocol was registered under the Oxford University Hospitals NHS Trust requirements (registration number 8723). Data were collected systematically regarding the clinicopathological characteristics, treatment intervention, and follow-up.

### 2.2. Data Collection

The design, analysis, interpretation of data, as well as the drafting and revisions, conform to the Helsinki Declaration and the Committee on Publication Ethics guidelines (http://publicationethics.org/, accessed on 13 December 2023) [18]. The reporting of studies was conducted using the observational routinely collected health data (RECORD) Statement, validated by the Enhancing the Quality and Transparency of Health Research Network (www.equator-network.org, accessed on 13 December 2023) [19]. The data collected were anonymized, considering the observational nature of the study, without personal data that could lead to the formal identification of the patient. Patients’ demographics and comorbidities were recorded. An age-adjusted Charlson comorbidities score was used to stratify comorbidities [20]. The nature of surgery and any adjuvant therapy received was also extracted, along with the duration of postoperative hospital stay and complications. The postoperative complications were classified according to Clavien–Dindo classification [21]. Overall survival (OS) was defined as the time (months) from the initiation of surgery/diagnosis (if too advanced to offer surgery) to death from any cause. Disease-free survival (DFS) was measured in patients declared disease-free from the date of primary surgery to the date of disease recurrence. Progression-free survival (PFS) was measured from the date of primary surgery to the date of diagnosis of disease progression. The cut-off point for the survival study was 30 October 2023. Follow-up was recorded with recurrences, and treatment modality used to manage recurrences.

### 2.3. Endpoints and Outcomes

The main outcomes measured were the survival and recurrence patterns. The management and follow-up strategy were also analyzed. Immunohistochemical and molecular analyzes, wherever performed, were also analyzed. The main aims of the study were to evaluate the survival outcomes and efficiency of the follow-up regimes used.

### 2.4. Statistical Analysis

The data were analyzed using IBM SPSS 28 and Jamovi version 2.3.28.0. Mean data with standard deviation were calculated for descriptive data. The survival rates were calculated by the Kaplan–Meier method, and those variables were evaluated by univariate analysis. The multivariate analysis used to identify correlations between our findings and all potential parameters involved using a logistic regression model. The comparison of survival curves between groups was performed using the log-rank test. A *p* value less than 0.05 was significant for all tests.

## 3. Results

### 3.1. Demographic Data

A total of 41 patients had histology-proven uterine sarcoma, classified as LMS, ADS and ESS, respectively, in 25, 8, and 8 cases. In the ESS group, HG-ESS and LG-ESS were, respectively, 5 and 3. The mean age at diagnosis was 56.8 years (range 34–82 years), and most were postmenopausal (61%). The mean BMI was 29.73, and median follow-up was 51.4 months.

The clinical presentation in 41% patients was symptoms related to a pelvic mass, with vaginal bleeding being the second most frequent symptom (34%). In six patients, the diagnosis was made postoperatively on histological examination of a presumed benign fibroid. Table 1 depicts a summary of the clinical characteristics of the women enrolled in the study.

### 3.2. Management and Follow-Up for Leiomyosarcoma

All patients were discussed at a multidisciplinary conference before and after primary treatment.

In 68% of the cases, the patients underwent surgery (n = 17). A total abdominal hysterectomy with bilateral salpingo-oophorectomy was the commonest operation performed, and 20% had consensual omentectomy or omental biopsy. Only one patient had the resection of pelvic and paraaortic lymph nodes. A 34-year-old nulliparous patient with Stage I low-grade leiomyosarcoma had subtotal abdominal hysterectomy, and unilateral salpingo-oophorectomy, and LMS was diagnosed only postoperatively on histology, and so a subsequent surgery was performed. Only one woman underwent laparoscopic morcellation during subtotal hysterectomy procedure (performed for a presumed benign cause in the view of heavy prolonged menstrual bleeding).

**Figure 1:**
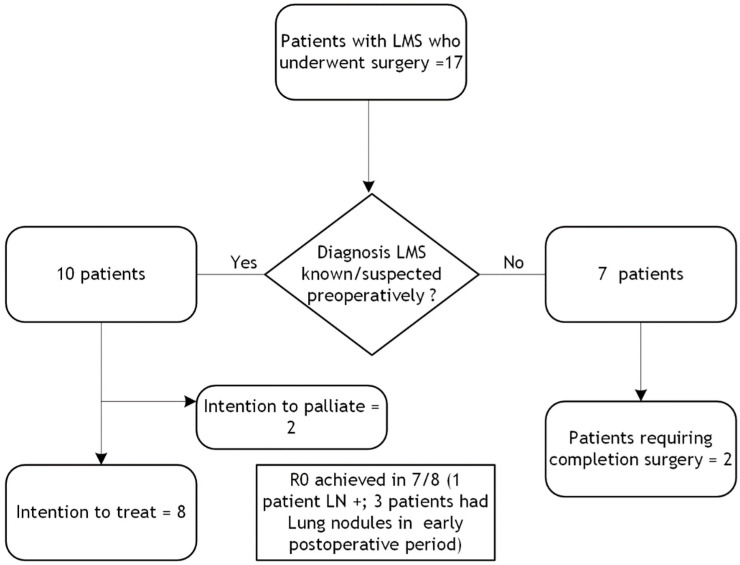
Distribution of patients managed surgically (LMS = leiomyosarcoma; LN = lymph node).

Where LMS was suspected preoperatively, surgeries were mostly performed with the intention to treat except in two cases, as can be seen in Figure 1. All except one patient achieved optimal cytoreduction at the time of surgery, which was confirmed at histology. Two patients had palliative surgery due to abdominal symptoms.

Four patients had grade II complications, while only one patient had a grade IIIa complication. The average hospital stay after surgical management was 5.2 days. Ten (59%) received adjuvant chemotherapy. The agents used were Gemcitabine, Docetaxel or Doxorubicin, and Ifosfamide, with an average of 3.5 cycles of therapy for each case. One woman had external beam radiotherapy (EBRT) after a partial response to Gemcitabine and Docetaxel.

Eight patients (32%) were considered unsuitable for surgical management due to advanced stage. Four patients had chemotherapy, and one had palliative EBRT. Trabectedin was used in three patients after careful consideration. Based on the hormone receptor status, adjuvant hormone therapy was used in 88% (n = 7) of the cases, and letrozole was the most common hormone therapy used, with Goserelin used in only one woman.

**Figure 2:**
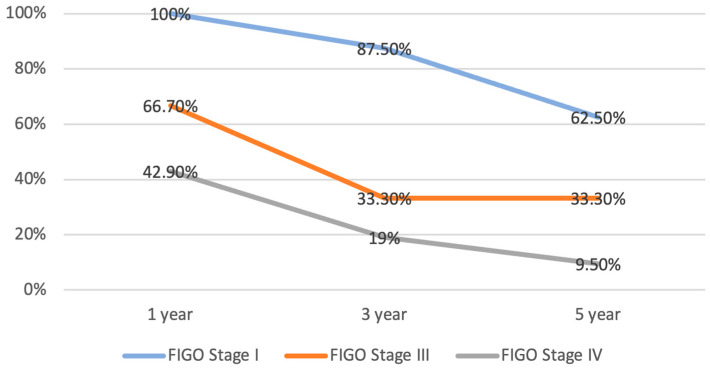
One-, three- and five-year OS for LMS, according to stage (OS = overall survival; LMS = leiomyosarcoma).

The mean disease-free survival (DFS) was 89.6 months among the patients declared disease-free, after the first curative intent treatment (n = 6). The mean PFS in for the patient who did not achieve complete response was 11.4 months (n = 19). The median overall survival OS was 25.2 months (n = 25). The percentage survival according to FIGO stage is depicted in Figure 2.

Immunohistochemical (IHC) markers were analyzed among the tumor specimens. Most tumor specimens showed positivity for desmin, smooth muscle actin (SMA), vimentin, and caldesmon. Other histology and IHC characteristics are denoted in Table 2. Hormone receptor positivity was also found in 40% of tumors for estrogen, and 32% of tumors for progesterone. p53 mutation was detected in 20% of tumor specimens analyzed. A molecular analysis report was only available for one patient with respect to whole-genome sequencing, which predicted ATRX loss and signature 3 (homologous recombination deficiency) positivity with the loss of TP53 and RB1.

All patients had intensive follow-up. They were reviewed clinically, and a CT (Computed tomographic) scan of the chest, abdomen, and pelvis was performed at a frequency of 3–6 months.

Nineteen patients failed to achieve disease-free status and showed progression, while six patients achieved disease-free status. All six patients who achieved disease-free status had the recurrence of disease. The most common site of recurrence was the pelvis (66.6%), followed by the lungs (33.3%). All the recurrences were detected either via the treatment monitoring or surveillance imaging. The majority of recurrences (83.3%) were treated surgically. Patients with pelvic recurrences underwent resection, with one patient undergoing posterior exenteration surgery, and another one had anterior resection. Lung recurrences were managed surgically with the involvement of thoracic surgery team. All recurrences were confirmed on the histology examination. Chemotherapy and hormone adjuvant therapy were also used in patients with recurrences who were not suitable for surgery.

### 3.3. Management and Follow-Up for Adenosarcoma

ADS patients presented with vaginal bleeding in all the cases (n = 8) and all of them underwent primary surgical management. Seven of these patients were preoperatively diagnosed with ADS. Minimally invasive surgery was the preferred route (75%). All patients had low-grade disease with the mean tumor size of 22.1 mm (range 13–52 mm) at FIGO Stage I. Four patients had positivity for ER, PR, and CD 10.

No patient required adjuvant therapy, and to date there have been no recurrences with a mean follow-up time of 53 months (range 12.1–94.6) and a median OS of 52.7 months. The commonest regime used was a CT scan of the abdomen and pelvis performed at 6, 12, and 18 months. A chest X-ray was performed every three months for 2 years, every 6 months for years 3–5, and then annually for years 5–10.

### 3.4. Management and Follow-Up for Endometrial Stromal Sarcoma

Five patients (63%) in the ESS group had surgical management, while the rest were not considered suitable for surgery. FIGO stage I was the most common stage, and minimally invasive surgery was the preferred surgical route. Four patients had total laparoscopic hysterectomy and bilateral salpingo-oophorectomy. Two patients had radical debulking surgery. The mean tumor size was 55.4 mm (range 25–70 mm). One patient had debulking surgery with no residual disease, and received adjuvant chemotherapy (Doxorubicin), while two patients received EBRT. SMA positivity was demonstrated in 75% of the cases on IHC, while CD10 positivity was seen in 50% of them. Mutant p53 was found in only one case, while estrogen receptor positivity was only seen in 25% of the cases. Only one patient received anastrozole as an adjuvant therapy.

The surveillance protocol was similar to the LMS group, with imaging and clinical follow-up every 3–6 months. Four patients achieved disease-free status (50%), and the mean DFS was 26.34 months. Two patients had recurrences: one had a pelvic recurrence, which was surgically resected, while another patient developed a rapidly progressing rib metastasis six months after surgery. The mean PFS was 6.3 months. The median OS for the ESS group was 11.8 months. Table 3 summarizes the treatment modalities used in all three patient groups, and the related outcomes.

### 3.5. Survival Analysis

**Figure 3:**
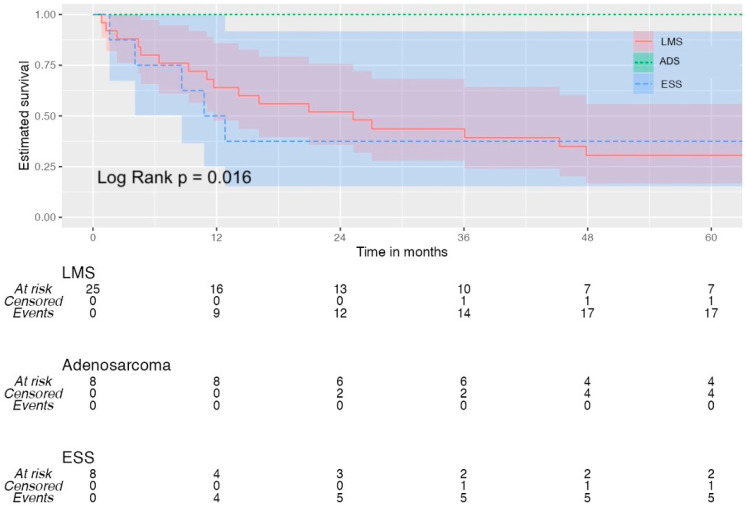
Overall survival for all patients. (LMS = leiomyosarcoma; ADS = adenosarcoma; ESS = endometrial stromal sarcoma).

**Figure 4:**
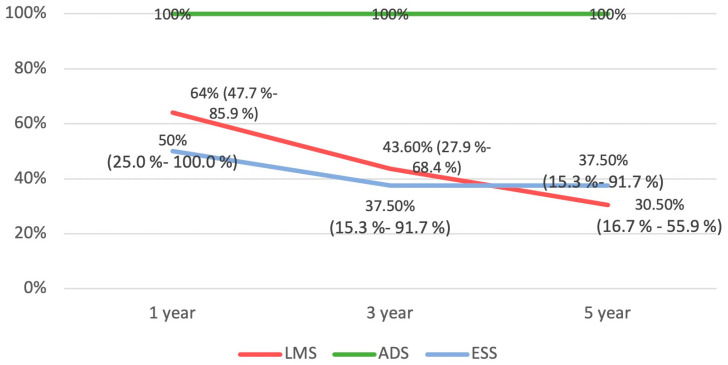
Survival rates at one, three, and five years with 95% confidence interval (reported where applicable). (LMS = leiomyosarcoma; ADS = adenosarcoma; ESS = endometrial stromal sarcoma).

The Kaplan–Meier survival analysis plotted for all three groups is available in Figure 3 and Figure 4. The ADS group had a significantly better OS compared to the LMS and ESS groups (*p* = 0.016).

## 4. Discussion

Uterine sarcomas account for less than 7% uterine cancers, and the worldwide incidence is about 1.7 in 100,000 women [22]. To date, the data available have been mostly from studies constituted by small numbers of patients. We present our experience with these tumors at a tertiary referral center with a considerable sample size over ten years. LMS was the most common histology type among the patient cohort with a median OS of 25.2 months.

### 4.1. General Characteristics of Uterine Sarcomas

The mean age at diagnosis in our study was 56.8 years, which is in keeping with the rest of the available literature, including a large study by Ruiz-Minaya et al. [23]. Once diagnosed with sarcoma, which was sometimes a postoperative diagnosis, these patients were managed as per the guidance for the management of uterine sarcoma. All these patients were discussed at a multidisciplinary conference, promptly after the histology report of uterine sarcoma. Casarin et al. studied incidentally discovered uterine leiomyosarcoma in 5528 consecutive hysterectomies, and they found no significant difference in survival between patients with preoperative suspected or confirmed disease versus those with incidentally uterine LMS. There are many studies showing the adverse impact of tumor fragmentation in uterine LMS on patterns of recurrence and prognostic outcomes [24,25], and as we only encountered one woman having morcellation at the primary procedure, the impact of the morcellation on the prognosis could not be studied.

LMS was the most prevalent histological subtype in our patient cohort (60.9%). This concurs with other publications [26,27,28,29]. The immunohistochemical profiling used in our population was consistent with findings in other studies.

Immunohistochemical staining with smooth muscle actin, desmin, and caldesmon show the positive reactions in tumor cells, and p53, p16, estrogen, progesterone, and ki-67 help to differentiate uterine sarcoma from benign leiomyoma. There are no immunohistochemical markers pathognomonic for adenosarcoma. The most common ones used are CD10, ER, PR, AE1/AE3, vimentin, actin, and WT1 [30]. LG-ESS tumor cells are strongly positive for CD10. Smooth muscle actin and caldesmon are positive in areas with smooth muscle differentiation, whereas inhibin, calretinin, melan-A, and CD99 are usually positive in rare areas with sex cord-like differentiation [31]. Surgery is recognized as the mainstay of treatment in early-stage sarcoma, as in this study [32]. The single report of WGS that we had access to shows molecular characteristics in line with the current evidence. It is supposed that chromosomal instabilities (complex numerical and structural chromosomal aberrations) are the hallmark of uterine smooth muscle tumors, but none are diagnostic. The most frequently mutated gene is p53 (30%), followed by alpha thalassemia/mental retardation syndrome X-linked (ATRX) (25%), mediator complex subunit 12 (MED12) (20%), retinoblastoma protein (RB1), and breast cancer gene-2 (BRCA2) [7,33,34,35].

The impact and role of adjuvant therapies in this condition remains unclear, even in high-risk patients [36,37,38,39]. In this study, those who received adjuvant chemotherapy were under the ESMO, EURACAN, and GENTURIS guidelines [32]. Trabectedin was used in some patients, in line with various studies showing its effect on the quality of life and long-term stabilization of the tumor [40]. Hormone therapy was also used based on the hormone receptor status of the tumor [41].

### 4.2. Follow-Up Regimens

The optimum form of follow-up and use of imaging is variable in this group of sarcomas. The typical surveillance protocol suggested for uterine sarcomas is CT scanning of the chest, abdomen, and pelvis and this is recommended every 3 to 6 months for the first three years, and then every 6 to 12 months for the following two years [39,42]. Our surveillance protocol for LMS was like this, with clinical review and imaging every 3–6 months. In our population, there was a 90% pick rate for recurrences; 10% were driven by patient symptoms outside the planned follow-up regimen.

In our study, ADS had the best prognosis in terms of DFS and OS, as reported by others [43,44,45,46,47,48]. The main presenting symptom of vaginal bleeding and diagnosis at stage I has previously been reported [49]. The optimum follow-up strategy for these sarcomas is unclear. The approach we used was chest X-ray, performed every three months for 2 years, every 6 months for years 3–5, and then annually for years 5–10. There were no recurrences among the ADS group in our study [50].

### 4.3. Limitations of This Study

As this was a single-center study, we recognize that the main limitation of this study design was the relatively small numbers, especially of the non-leiomyosarcoma patients. In the ADS group, all the patients had low-grade disease and therefore, that could have potentially skewed the findings. Most of the patients in the ESS group had advanced high-grade ESS. In our study, we had insufficient cases of low-grade and high-grade ESS to compare these two pathologies conclusively. We also did not have access to molecular analysis of these tumors, as the technology was only recently made accessible for patient population outside of approved research setting. As access to molecular analysis was not available for our patients before 2021, we could not analyze the molecular characteristics of most of our patient population.

There is a need for future collaborative study and/or prospective database in the UK for these rare tumors.

### 4.4. Future Considerations

A deeper comprehension of tumor biology and the development of prospective clinical trials based on molecular results are prerequisites for the advancement of LMS treatment. In patients with uterine sarcomas, prospective genomic profiling can help with diagnosis accuracy and guide treatment decisions. A study analyzing the mutational profile of 216 cases of LMS confirmed that the most frequent alterations involve TP53, RB1, and ATRX, and shows that PTEN mutations tend to be acquired at thev advanced disease stages [51]. Homozygous deletions of BRCA2 were present in 5% of uterine sarcoma patients in another study and evidence of clinical benefit in patients with uterine leiomyosarcoma with somatic BRCA2 alterations treated with PARP inhibitors has been described [52].

## 5. Conclusions

Even though this retrospective study has a limited sample size because of the rarity of this tumor, our study presents important real-world data from a tertiary center. Uterine sarcomas are recognized to be aggressive tumors, and our study observed a significant association between tumor histological types, tumor stage, and OS. Surgical management remains the cornerstone of treatment of these tumors and was utilized as the main treatment modality in patients suitable for surgery in this study. The optimum follow-up strategies are unclear, but the management and follow-up of each subtype need to be tailored accordingly after thorough patient-focused multidisciplinary discussion.

## Figures and Tables

**Table 1:** Clinical characteristic.

	All Uterine Sarcomas (n = 41)	LMS (n = 25)	ADS (n = 8)	ESS (n = 8)
**Age (mean ± SD)**	56.82 (±13.12)	53.76 (±12.45)	54.75 (±9.08)	68.50 (±12.02)
**Charlson comorbidity Index**				
0–3	16 (39%)	8 (32%)	6 (75%)	2 (25%)
4–6	16 (39%)	10 (40%)	2 (25%)	4 (50%)
>7	9 (21.9%)	7 (28%)	0	2 (25%)
**Postmenopausal**	25 (61%)	12 (48%)	6 (75%)	8 (100%)
**FIGO Stage**				
Stage I		8 (32%)	8 (100%)	4 (50%)
Stage II		0	0	0
Stage III		3 (12%)	0	1 (12.5%)
Stage IV		14 (56%)	0	3 (37.5%)
**BMI (mean ± SD)**	29.7 (±7.037)	30.1 (±6.35)	26.12 (±3.21)	32.12 (±9.89)
**Vaginal bleeding**	20 (34.1%)	6 (24%)	8 (100%)	6 (75%)
**Bulking pelvic mass**	17 (41.4%)	16 (64%)	-	1 (12.5%)
**Systemic symptoms**	-	3 (12%)	-	-
**Other symptoms**	-	-	-	1 (12.5%)

Reported as number (percentage of group sample) unless specified that reported as mean, LMS = leiomyosarcoma; ADS = adenosarcoma; ESS = endometrial stromal sarcoma; SD = standard deviation; FIGO = International Federation of Gynecology and Obstetrics; BMI = Body Mass Index.

**Table 2:** Histological characteristics of LMS group.

Histological/IHC Characteristics	Percentage of Sample Expressing the Characteristic
Desmin	68%
Smooth muscle actin	84%
Vimentin	64%
Caldesmon	48%
Ki 67 proliferation index high	40%
p16	32%
CD10	20%
p53	20%
WT-1	8%
Necrosis	60%
Mitosis >/= 30/100HPF	28%
Mitosis counts 15–30/100HPF	68%
Average tumor size *	133 mm (30–250 mm)
ER-positive	40%
PR-positive	32%

IHC = immunohistochemistry; HPF = High Power Field; ER = estrogen receptor; PR = progesterone receptor; * = reported as mean and range in parathesis.

**Table 3:** Treatment modalities and outcomes.

	All Uterine Sarcomas (n = 41)	LMS (n = 25)	ADS (n = 8)	ESS (n = 8)
**Treatment modality used**				
**Surgery alone**	20 (48.7%)	7 (28%)	8 (100%)	5 (62.5%)
**Surgery and adjuvant CT**	11 (26.8%)	10 (40%)	0	1 (12.5%)
Adjuvant CT modality *				
Single drug adjuvant CT	2 (4.8%)	1 (4%)	0	1 (1.2%)
Multi drug adjuvant CT	9 (21.9%)	9 (36%)	0	0
**Palliative CT**	7 (17.1%)	7 (28%)	0	0
**Surgery and adjuvant EBRT**	1 (2.4%)	0	0	1 (1.2%)
**Palliative radiotherapy**	2 (4.8%)	1 (4%)	0	1 (1.2%)
**Disease-free patients**	18 (43.9%)	6 (24%)	8 (100%)	4 (50%)
Recurrences **	8 (44.4%)	6 (100%)	0	2 (50%)

Data are reported as number (%); LMS = leiomyosarcoma; ADS = adenosarcoma; ESS = endometrial stromal sarcoma; CT = chemotherapy; EBRT = external beam radiotherapy. * further detail of adjuvant chemotherapy used in the row above; ** denominator used = disease-free patients in the group.

## Data Availability

Data available on request.

## References

[1] D’Angelo E., Prat J. (2010). Uterine Sarcomas: A Review. Gynecol. Oncol..

[2] Parker W.H. (2005). Uterine Myomas: An Overview of Development, Clinical Features, and Management. Obstet. Gynecol..

[3] Brooks S.E., Zhan M., Cote T., Baquet C.R. (2004). Surveillance, Epidemiology, and End Results Analysis of 2677 Cases of Uterine Sarcoma 1989–1999. Gynecol. Oncol..

[4] Major F.J., Blessing J.A., Silverberg S.G., Morrow C.P., Creasman W.T., Currie J.L., Yordan E., Brady M.F. (1993). Prognostic Factors in Early-stage Uterine Sarcoma: A Gynecologic Oncology Group Study. Cancer.

[5] Mbatani N., Olawaiye A.B., Prat J. (2018). Uterine Sarcomas. Int. J. Gynaecol. Obstet.

[6] Hanby A.M., Walker C. (2004). Tavassoli FA, Devilee P: Pathology and Genetics: Tumours of the Breast and Female Genital Organs. WHO Classification of Tumours Series-Volume IV. Lyon, France: IARC Press. Breast Cancer Res..

[7] Cree I.A., White V.A., Indave B.I., Lokuhetty D. (2020). Revising the WHO Classification: Female Genital Tract Tumours. Histopathology.

[8] Parkash V., Aisagbonhi O., Riddle N., Siddon A., Panse G., Fadare O. (2023). Recent Advances in the Classification of Gynecological Tract Tumors: Updates from the 5th Edition of the World Health Organization “Blue Book”. Arch. Pathol. Lab. Med..

[9] Santos P., Cunha T.M. (2015). Uterine Sarcomas: Clinical Presentation and MRI Features. Diagn. Interv. Radiol..

[10] Puliyath G., Nair M.K. (2012). Endometrial Stromal Sarcoma: A Review of the Literature. Indian J. Med. Paediatr. Oncol..

[11] Borella F., Mancarella M., Preti M., Mariani L., Stura I., Sciarrone A., Bertschy G., Leuzzi B., Piovano E., Valabrega G. (2023). Uterine Smooth Muscle Tumors: A Multicenter, Retrospective, Comparative Study of Clinical and Ultrasound Features. Int. J. Gynecol. Cancer.

[12] Ludovisi M., Moro F., Pasciuto T., Di Noi S., Giunchi S., Savelli L., Pascual M.A., Sladkevicius P., Alcazar J.L., Franchi D. (2019). Imaging in Gynecological Disease (15): Clinical and Ultrasound Characteristics of Uterine Sarcoma. Ultrasound Obstet. Gynecol..

[13] Tropé C.G., Abeler V.M., Kristensen G.B. (2012). Diagnosis and Treatment of Sarcoma of the Uterus. A Review. Acta Oncol..

[14] Ghirardi V., Bizzarri N., Guida F., Vascone C., Costantini B., Scambia G., Fagotti A. (2019). Role of Surgery in Gynaecological Sarcomas. Oncotarget.

[15] Cantú De León D., González H., Pérez Montiel D., Coronel J., Pérez-Plasencia C., Villavicencio-Valencia V., Soto-Reyes E., Herrera L.A. (2013). Uterine Sarcomas: Review of 26 Years at The Instituto Nacional de Cancerologia of Mexico. Int. J. Surg..

[16] Giuntoli R.L., Garrett-Mayer E., Bristow R.E., Gostout B.S. (2007). Secondary Cytoreduction in the Management of Recurrent Uterine Leiomyosarcoma. Gynecol. Oncol..

[17] Oliva E. (2014). Cellular Mesenchymal Tumors of the Uterus: A Review Emphasizing Recent Observations. Int. J. Gynecol. Pathol..

[18] World Medical Association (2013). World Medical Association World Medical Association Declaration of Helsinki: Ethical Principles for Medical Research Involving Human Subjects. JAMA.

[19] Benchimol E.I., Smeeth L., Guttmann A., Harron K., Moher D., Petersen I., Sørensen H.T., Von Elm E., Langan S.M. (2015). RECORD Working Committee The REporting of Studies Conducted Using Observational Routinely-Collected Health Data (RECORD) Statement. PLoS Med..

[20] Charlson M.E., Pompei P., Ales K.L., MacKenzie C.R. (1987). A New Method of Classifying Prognostic Comorbidity in Longitudinal Studies: Development and Validation. J. Chronic. Dis..

[21] Clavien P.A., Barkun J., de Oliveira M.L., Vauthey J.N., Dindo D., Schulick R.D., de Santibañes E., Pekolj J., Slankamenac K., Bassi C. (2009). The Clavien-Dindo Classification of Surgical Complications: Five-Year Experience. Ann. Surg..

[22] Amant F., Coosemans A., Debiec-Rychter M., Timmerman D., Vergote I. (2009). Clinical Management of Uterine Sarcomas. Lancet Oncol..

[23] Ruiz-Minaya M., Mendizabal-Vicente E., Vasquez-Jimenez W., Perez-Burrel L., Aracil-Moreno I., Agra-Pujol C., Bernal-Claverol M., Martínez-Bernal B.L., Muñoz-Fernández M., Morote-Gonzalez M. (2022). Retrospective Analysis of Patients with Gynaecological Uterine Sarcomas in a Tertiary Hospital. J. Pers. Med..

[24] Bogani G., Cliby W.A., Aletti G.D. (2015). Impact of Morcellation on Survival Outcomes of Patients with Unexpected Uterine Leiomyosarcoma: A Systematic Review and Meta-Analysis. Gynecol. Oncol..

[25] Ricci S., Stone R.L., Fader A.N. (2017). Uterine Leiomyosarcoma: Epidemiology, Contemporary Treatment Strategies and the Impact of Uterine Morcellation. Gynecol. Oncol..

[26] Nano O., Nieto M.J., Saif M.W., Tarabichi M. (2020). Retrospective Analysis of Patients with Gynecological Uterine Sarcomas: Leiomyosarcomas and Other Histological Subtypes at a Single Institution from 1996 to 2015. Cancer Med. J..

[27] Kapp D.S., Shin J.Y., Chan J.K. (2008). Prognostic Factors and Survival in 1396 Patients with Uterine Leiomyosarcomas: Emphasis on Impact of Lymphadenectomy and Oophorectomy. Cancer.

[28] Kyriazoglou A., Liontos M., Ziogas D.C., Zagouri F., Koutsoukos K., Tsironis G., Tsiara A., Kaparelou M., Zakopoulou R., Thomakos N. (2018). Management of Uterine Sarcomas and Prognostic Indicators: Real World Data from a Single-Institution. BMC Cancer.

[29] Abeler V.M., Røyne O., Thoresen S., Danielsen H.E., Nesland J.M., Kristensen G.B. (2009). Uterine Sarcomas in Norway. A Histopathological and Prognostic Survey of a Total Population from 1970 to 2000 Including 419 Patients. Histopathology.

[30] Kostov S., Kornovski Y., Ivanova V., Dzhenkov D., Metodiev D., Watrowski R., Ivanova Y., Slavchev S., Mitev D., Yordanov A. (2021). New Aspects of Sarcomas of Uterine Corpus—A Brief Narrative Review. Clin. Pract..

[31] Horng H.-C., Wen K.-C., Wang P.-H., Chen Y.-J., Yen M.-S., Ng H.-T. (2016). Taiwan Association of Gynecology Systematic Review Group Uterine Sarcoma Part II-Uterine Endometrial Stromal Sarcoma: The TAG Systematic Review. Taiwan J. Obstet. Gynecol..

[32] Gronchi A., Miah A.B., Dei Tos A.P., Abecassis N., Bajpai J., Bauer S., Biagini R., Bielack S., Blay J.Y., Bolle S. (2021). Soft Tissue and Visceral Sarcomas: ESMO–EURACAN–GENTURIS Clinical Practice Guidelines for Diagnosis, Treatment and Follow-Up☆. Ann. Oncol..

[33] Cui R.R., Wright J.D., Hou J.Y. (2017). Uterine Leiomyosarcoma: A Review of Recent Advances in Molecular Biology, Clinical Management and Outcome. BJOG.

[34] Yang C.-Y., Liau J.-Y., Huang W.-J., Chang Y.-T., Chang M.-C., Lee J.-C., Tsai J.-H., Su Y.-N., Hung C.-C., Jeng Y.-M. (2015). Targeted Next-Generation Sequencing of Cancer Genes Identified Frequent TP53 and ATRX Mutations in Leiomyosarcoma. Am. J. Transl. Res..

[35] An Y., Wang S., Li S., Zhang L., Wang D., Wang H., Zhu S., Zhu W., Li Y., Chen W. (2017). Distinct Molecular Subtypes of Uterine Leiomyosarcoma Respond Differently to Chemotherapy Treatment. BMC Cancer.

[36] Bogani G., Fucà G., Maltese G., Ditto A., Martinelli F., Signorelli M., Chiappa V., Scaffa C., Sabatucci I., Lecce F. (2016). Efficacy of Adjuvant Chemotherapy in Early Stage Uterine Leiomyosarcoma: A Systematic Review and Meta-Analysis. Gynecol. Oncol..

[37] Roque D.R., Taylor K.N., Palisoul M., Wysham W.Z., Milam B., Robison K., Gehrig P.A., Raker C., Kim K.H. (2016). Gemcitabine and Docetaxel Compared with Observation, Radiation, or Other Chemotherapy Regimens as Adjuvant Treatment for Stage I-to-IV Uterine Leiomyosarcoma. Int. J. Gynecol. Cancer.

[38] Bacalbasa N., Balescu I., Dima S., Brasoveanu V., Popescu I. (2015). Prognostic Factors and Survival in Patients Treated Surgically for Primary and Recurrent Uterine Leiomyosarcoma: A Single Center Experience. Anticancer Res..

[39] Koh W.-J., Greer B.E., Abu-Rustum N.R., Apte S.M., Campos S.M., Cho K.R., Chu C., Cohn D., Crispens M.A., Dizon D.S. (2015). Uterine Sarcoma, Version 1.2016: Featured Updates to the NCCN Guidelines. J. Natl. Compr. Canc. Netw..

[40] Rubio M.J., Lecumberri M.J., Varela S., Alarcón J., Ortega M.E., Gaba L., Espinós J., Calzas J., Barretina P., Ruiz I. (2020). Efficacy and Safety of Trabectedin in Metastatic Uterine Leiomyosarcoma: A Retrospective Multicenter Study of the Spanish Ovarian Cancer Research Group (GEICO). Gynecol. Oncol. Rep..

[41] Hardman M.P., Roman J.J., Burnett A.F., Santin A.D. (2007). Metastatic Uterine Leiomyosarcoma Regression Using an Aromatase Inhibitor. Obstet. Gynecol..

[42] Roy M., Musa F., Taylor S.E., Huang M. (2022). Uterine Sarcomas: How to Navigate an Ever-Growing List of Subtypes. Am. Soc. Clin. Oncol. Educ. Book.

[43] Wang Q., Sun S., Cai J., Yang L., Lv G., Yang Q. (2023). Uterine Adenosarcoma: A Case Report and Review of the Literature. Am. J. Nucl. Med. Mol. Imaging.

[44] Arend R., Bagaria M., Lewin S.N., Sun X., Deutsch I., Burke W.M., Herzog T.J., Wright J.D. (2010). Long-Term Outcome and Natural History of Uterine Adenosarcomas. Gynecol. Oncol..

[45] Bernard B., Clarke B.A., Malowany J.I., McAlpine J., Lee C.-H., Atenafu E.G., Ferguson S., Mackay H. (2013). Uterine Adenosarcomas: A Dual-Institution Update on Staging, Prognosis and Survival. Gynecol. Oncol..

[46] Friedlander M.L., Covens A., Glasspool R.M., Hilpert F., Kristensen G., Kwon S., Selle F., Small W., Witteveen E., Russell P. (2014). Gynecologic Cancer InterGroup (GCIG) Consensus Review for Mullerian Adenosarcoma of the Female Genital Tract. Int. J. Gynecol. Cancer.

[47] Ulrich U.A., Denschlag D. (2018). Uterine Adenosarcoma. Oncol. Res. Treat..

[48] Pinto A., Howitt B. (2016). Uterine Adenosarcoma. Arch. Pathol. Lab. Med..

[49] Shi Y., Liu Z., Peng Z., Liu H., Yang K., Yao X. (2008). The Diagnosis and Treatment of Mullerian Adenosarcoma of the Uterus. Aust. NZJ Obstet. Gynaecol..

[50] Sundar S., Balega J., Crosbie E., Drake A., Edmondson R., Fotopoulou C., Gallos I., Ganesan R., Gupta J., Johnson N. (2017). BGCS Uterine Cancer Guidelines: Recommendations for Practice. Eur. J. Obstet. Gynecol. Reprod. Biol..

[51] Astolfi A., Nannini M., Indio V., Schipani A., Rizzo A., Perrone A.M., De Iaco P., Pirini M.G., De Leo A., Urbini M. (2020). Genomic Database Analysis of Uterine Leiomyosarcoma Mutational Profile. Cancers.

[52] Hensley M.L., Chavan S.S., Solit D.B., Murali R., Soslow R., Chiang S., Jungbluth A.A., Bandlamudi C., Srinivasan P., Tap W.D. (2020). Genomic Landscape of Uterine Sarcomas Defined Through Prospective Clinical Sequencing. Clin. Cancer Res..

